# A qualitative investigation of the role of sport coaches in designing and delivering a complex community sport intervention for increasing physical activity and improving health

**DOI:** 10.1186/s12889-018-6089-y

**Published:** 2018-10-22

**Authors:** Louise Mansfield, Tess Kay, Nana Anokye, Julia Fox-Rushby

**Affiliations:** 1 Department of Life Sciences, Brunel University London, Kingston Lane, Uxbridge, Middlesex, UB8 3PH UK; 2 Department of Clinical Sciences, Brunel University London, Kingston Lane, Uxbridge, Middlesex, UB8 3PH UK; 3 Department of Primary Care and Public Health Sciences, King’s College London, Addison House, Guy’s, London, SE1 1UL UK

**Keywords:** Community sport, Complex community intervention, Sport coaches, Public health, Physical activity

## Abstract

**Background:**

Community sport can potentially help to increase levels of physical activity and improve public health. Sport coaches have a role to play in designing and implementing community sport for health. To equip the community sport workforce with the knowledge and skills to design and deliver sport and empower inactive participants to take part, this study delivered a bespoke training package on public health and recruiting inactive people to community sport for sport coaches. We examined the views of sport coach participants about the training and their role in designing and delivering a complex community sport intervention for increasing physical activity and improving health.

**Methods:**

Semi-structured interviews were conducted with paid full-time sport coaches (*n* = 15) and community sport managers and commissioners (*n* = 15) with expertise in sport coaching. Interviews were conducted by a skilled interviewer with in-depth understanding of community sport and sport coach training, transcribed verbatim and analysed using thematic analysis.

**Results:**

Three key themes were identified showing how the role of sport coaches can be maximised in designing and delivering community sport for physical activity and health outcomes, and in empowering participants to take part. The themes were: (1) training sport coaches in understanding public health, (2) public involvement in community sport for health, and (3) building collaborations between community sport and public health sectors.

**Conclusion:**

Training for sport coaches is required to develop understandings of public health and skills in targeting, recruiting and retaining inactive people to community sport. Public involvement in designing community sport is significant in empowering inactive people to take part. Ongoing knowledge exchange activities between the community sport and public health sector are also required in ensuring community sport can increase physical activity and improve public health.

**Electronic supplementary material:**

The online version of this article (10.1186/s12889-018-6089-y) contains supplementary material, which is available to authorized users.

## Background

Regular physical activity is significant in the prevention and treatment of physical and mental health conditions including cardiovascular disease, diabetes, hypertension, osteoporosis, some cancers, anxiety and depression [1]. Worldwide, the prevalence of physical activity at recommended levels is low. Current estimates in the UK are that approximately 20 million adults (39%) are categorised as inactive because they fail to meet the recommended guidelines for physical activity of 150 min per week of moderate intensity physical activity and strength exercise on at least 2 days [2]. Increasing population levels of physical activity can potentially improve public health. In England, the Moving More, Living More cross Government group includes representation from national lead agencies, Sport England, the Department of Health and Public Health England and recognises the role that sport can play in helping people to become more active for improved health outcomes [3–5]. This perspective reflects more recent debates about the potential of low intensity physical activity for improving health which challenge established physical activity for health guidelines emphasising moderate and vigorous intensity phyiscal activity [6].

Successive Sport England strategies have focused on developing sporting opportunites tailored to the needs of diverse communities of local users. With devolvement of public health priorities to local authorities in April 2013, there is a heightened significance of locally based initiatives and the role of complex community interventions for public health outcomes; those that involve several interlocking components important to successful delivery [7, 8]. Community-centred interventions can have a positive impact on health behaviours [9, 10]. Successful community-based health interventions are associated with extensive formative research, participatory strategies and a theoretical and practical focus on changing social norms [11].

Sport coaches have a vital role to play in changing social norms around sports through individual and community engagement and empowering or enabling participants to take part in physical activity [12]. Empowerment theory provides a useful theoretical approach for understanding the complexities of raising physical activity levels through community sport. At the community level, empowerment theory investigates people’s capacity to influence organisations and institutions which impact on their lives [13, 14]. The theory addresses the processes by which personal and social factors of life enable and constrain behaviours, and this provides the theoretical basis of this study.

There are 1,109, 000 sport coaches in the UK primarily working in sports clubs or extra-curricular school-based programmes, with much of their expertise focused on beginners and learners and sport enthusiasts [15]. Sports coaches represent community assets in the development of sport for health programmes for inactive adults who may be apprehensive rather than enthusiastic about taking part in sport [12], yet little is known about the occupational drivers, priorities and requirements of this workforce. There is potential for them to be a resource for identifying and assessing inactive people and providing physical activity education, promotion and support in local public health environments; a role more commonly associated with routine care in GP surgeries and health centres [16, 17]. The potential of sports clubs as a health promotion setting has been recognised [18, 19]. Key issues have been identified in developing successful approaches to health promotion in sports clubs including the need for clear health focused strategies, adapting sports activity, ensuring a health promoting environment, enabling learning opportunities about sport for health and workforce training in public health [20]. Knowledge and skill development in the sport coach workforce is imperative to equip it to design, deliver and evaluate community sport opportunities for public health outcomes [21]. Most recent models for such workforce development advocate partnership approaches between sport and leisure providers, public health professionals and the participants for whom community sport programmes are designed and delivered [22]. The aim of this paper is to explore the role of sport coaches in designing and delivering a complex community sport intervention for increasing physical activity and improving health.

### Background to the study – The health and sport engagement (HASE) project

**Fig. 1:**
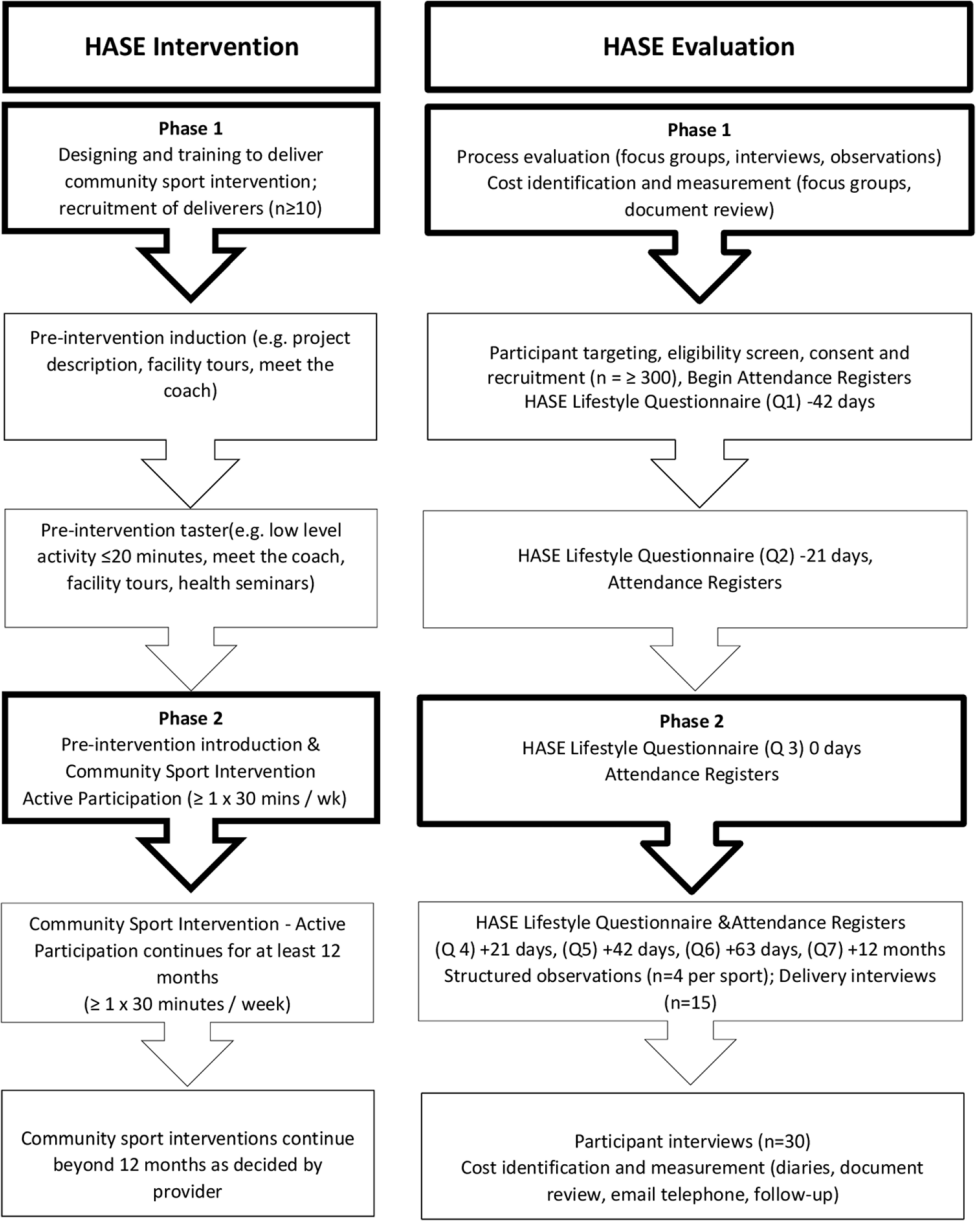
The Health and Sport Engagement (HASE) Study overview

Between March 2013 and July 2016, 32 sport coaches delivering and managing community sport in the London Borough of Hounslow were involved in a complex community sport intervention; the Health and Sport Engagement (HASE) project. The aim of the HASE project was to engage previously inactive people in sustained sporting activity for 1 × 30 min a week, examine the associated health and wellbeing outcomes of doing so, and produce information of value to those commissioning public health programmes that could potentially include sport. Full details of the HASE project are provided in the published protocol [23]. A summary of the HASE project intervention and evaluation phases is provided in Fig. 1.

Design and delivery of the HASE intervention involved a collaborative partnership between local community participants, sport coaches and community sport managers/ commissioners in the London Borough of Hounslow (LBH), and sport and public health researchers at Brunel University London. Coaches were key stakeholders in the project which employed a collaborative approach to stakeholder engagement, involving them in the initial project ideas development prior to the funding application, and in formative discussions about relevant training and the content and scheduling of training. Training served not only as a form of education and skill development but as a space for on-going involvement of coaches in the co-design [24] of the training programme, the precise nature of the sport activities and their delivery and evaluation approaches.

During a 12-month delivery phase, community sport coaches delivered 682 sport sessions to 550 people in the HASE project. Community sport coaches with expertise and experience in delivering and managing sport activities and with knowledge of diverse local communities were identified as central to the successful design and implementation of community sport for inactive people. A bespoke HASE training programme was included to identify existing expertise and additional skills and knowledge requirements of community sport coaches in designing and implementing community sport for health. The HASE training schedule for sport coaches consisted of two elements:

1. To develop understandings of public health for sport coaches, training included The Royal Society for Public Health (RSPH) Level 2 Award in Understanding Health Improvement, and workshops on targeting, promoting and retaining inactive people to sport (http://makesportfun.com/), and disability, inclusion and sport (https://disabilitysportscoach.co.uk/training-workshops/).
2. To address the need for cross sector collaboration and partnership between local sport and public health groups, sports coaches and public health professionals attended a bespoke knowledge exchange workshop on getting to know and understand the roles and working practices of personnel in each sector (http://makesportfun.com/).

Between March–September 2013, 32 community sport coaches were trained in the RSPH Level 2 Award in the first phase of the HASE project. Fifteen of those sport coaches were paid and full-time and they also engaged in training about targeting, recruiting and retaining inactive people in community sport and an on-line disability in sport course. Fourteen of those additionally attended knowledge exchange activities between sport coaches and public health professionals (1 coach was unavailable due to work commitments). Knowledge exchange activities included demonstrations of adapted sports activities, a ‘meet and greet’ event in which coaches and health professionals were paired to talk and exchange professional information, then paired with another expert at 5-min intervals, and a discussion forum about the strategy and mechanism of local authority public health referral scheme.

The HASE project included a mixed methods evaluation of the outcomes, processes and costs of the complex community sport intervention. Process evaluations are recommended in examining the efficacy of complex interventions and have value in multisite projects where the same interventions are tailored to the specific contexts and delivered and received in diverse ways [25]. Process evaluations using qualitative methods can complement research designs that assess effectiveness and efficiency quantitatively [26], by providing in-depth knowledge from those delivering and receiving the interventions. Evaluating the design, implementation, mechanisms of impact, and contextual factors that create different intervention effects can support the development of optimal complex community interventions and contribute to decision making about whether it is feasible to proceed to a larger scale trial [27]. This study presents findings from the interviews with sport coaches and community sport managers or commissioners with expertise in sport coaching which formed part of the process evaluation in the HASE project.

## Methods

### Data collection

Taking a pragmatic approach to evaluation to ensure timely, practice relevant yet rigorous research [28] the 15 sport coaches who had been trained in the RSPH Level 2 Award, attended the workshops and completed the on-line disability in sport course were invited for interview. All but one of those had also attended the knowledge exchange workshops with public health professionals. Fifteen community sport managers or commissioners with knowledge of sport coaching and involved in developing the HASE intervention and evaluation were also invited to interview. Thirty telephone interviews were conducted with paid full-time sport coaches (*n* = 15) and community sport managers and commissioners (*n* = 15) with expertise in sport coaching.

Semi structured interviews were conducted by one researcher (LM) for consistency of questioning. The aims of these interviews were twofold: (1) to examine the aspirations and logic underpinning design, delivery, promotion, and commissioning of sport for health projects, and (2) to examine the experiences and views of the HASE training. The interview guide can be found in Additional file 1. The interview data helped to determine the role of the sports coach in designing and delivering a complex community sport intervention for increasing physical activity and improving health. In this paper direct quotes are included in the results and respondents referred to by gender, self-reported job and coaching role and years of experience (YE).

### Analysis

Interviews were recorded and transcribed verbatim. Interview data were managed via NVivo 11 software and through the collation of tables and data matrices using Word 2010. The principles of thematic analysis were employed in this study. Thematic analysis allows the organisation, detailed description and scrutiny of patterns of meaning in qualitative data [29–31]. Analysis involved repeated reading, by two researchers (LM and TK), of interview transcripts, to determine the details of the data and to enable researchers to identify key themes and patterns in it. Themes were identified by theoretical approaches focused on our analytical interest in empowerment in community sport interventions, and by inductive (data-driven) approaches drawing directly from the data produced. Coding frameworks were devised by two researchers (LM and TK). Discrepancies were resolved by discussion and the codes and themes verified by all researchers (LM, TK, NA, JF-R) in a process of identifying, refining and interpreting key themes [32]. Anonymised quotes from interviewees are provided as evidence form our study. Punctuation was added to unambiguous quotes and where necessary, words added in parentheses to clarify intended meaning.

## Results

Interviewees had been employed in the community sport sector for between 6 months and 25 years. Interviews lasted between 18 and 50 min and the mean interview length was 26 min. Three key themes were identified from the interview data that illustrate the significance and role of the sport coach in designing and delivering community sport for physical activity and health outcomes, and in empowering participants to take part: (1) training sport coaches in understanding public health, (2) public involvement in community sport for health, and (3) building collaborations between community sport and public health. We present the results in sections to reflect the identified themes although we are mindful that the themes overlap.

### Training sport coaches in understanding public health

Phase 1 of the HASE project provided training for sport coaches and instructors to develop knowledge and understanding of public health and of targeting, recruiting and retaining inactive people to sport for health programmes. There was recognition amongst the HASE workforce of the potential for their work in community sport to support public health outcomes through the informal connections between health and their existing qualifications and experience:

*we’ve always recognised that it’s (sport) physical activity and health going hand in hand…..Yes this is about sport…but… it’s about engagement, it’s about physical activity, it’s about meaningful activity for young people and adults to gain confidence and skills, but actually it’s linked into health and healthy lifestyles as well (M,Community Sport Manager and Sport Coach, 15YE).*

The need for sports coaches to engage in training to develop their understanding of public health and their ability to deliver to health outcomes was also recognised. The training was delivered in two forms – an RSPH Level 2 award, and bespoke training workshops commissioned through the HASE project.

### Royal Society for public health (RSPH) level 2 award in understanding health improvement

The RSPH level 2 Award provided HASE sports coaches with the time and space to think about the relationships between sport and health and consider the significance of public health for their work. Those who participated expressed great enthusiasm for the training and emphasised that it had provided them with new knowledge and approaches that were highly relevant to their role in enabling people to become more active. The training was very highly valued:

*I’m really glad I took those courses…it changed how I did things…. especially the behaviour change parts …and the (health) things they encourage you to think about … with different groups… also the social and emotional aspect of that (physical activity and health)… it really helped …understand inactivity …and help people (M, Community Leader and Sport Coach, 5YE).*

*It just gave me some space to think about health…and how what I do can link to public health issues (M, Community Sport Coach, 5YE).*

A particularly important aspect of the delivery of the RSPH course was tailoring the information and subsequently the activities and their delivery to local population characteristics in Hounslow and to the requirements and priorities of the HASE workforce in supporting people to raise their physical activity levels:

*the Hounslow portion of that training was amazing, that was brilliant, I really liked it. I thought that it was crazy that people that live in Chiswick lived four years, on average, four years longer than people that live you know in like other parts of Hounslow for example. I can now talk to kids about health ..through sport (M, Community Sport Coach, 4YE).*

### Bespoke training workshops: Targeting, recruiting and retaining inactive participants

The workshops focusing on targeting, recruiting and retaining inactive people to sport gave sport coaches the knowledge and time to effectively plan their programmes:

*The workshops I thought were really good. I think when you’re actually discussing the practicalities, logistics, in reality how can we do this it’s definitely good to give you a chance to have discussions and actually properly sit down and plan. And I was able to go from one workshop, try something in the middle and then come back to the next one and talk about actually I did this and it worked. That sort of camaraderie in a way leaves you feeling motivated, ready to go. (F, Physical Activity Manager and Sport Coach Commissioner, 8YE).*

The workshops also helped sports coaches develop knowledge about best practice in supporting and engaging inactive groups in community sport:

*The qualification (RSPH) and those workshops are the right approach … they are about saying this is what we know now…this is the best way of doing it….it’s a forum where it brings people together where people meet on a course and then they’ve gone off and developed a programme together (M, Community Sport Commissioner, 15YE).*

*One of the key things was being in the mix with so many people from different sports... everyone had different stories to talk about, different experiences …knowledge …expertise to share (F, Community Coach Volunteer, 5YE).*

### Public involvement in community sport for health

Sport coaches identified the involvement of potential participants as important to the co-design and implementation of the community sports. Involving potential participants in designing their local sport offer was viewed as a way for sport coaches to identify both the physical activity and sporting needs of potential participants. It was also way for sport coaches to think about, understand and respond to practical barriers to participation but also the complex personal and social conditions, experiences and views that make taking part difficult; a key tenet of empowerment approaches:

*outreach….you’ve got to invest some time in it…..speaking to a captive audience …encouraging them and actually I think there was a desire, they did want to be active but the barrier was the transport and their own physical ability. So knowing that ….and having that barrier taken away from them, it was then easy to attract them in (F, Community Sports Development Manager and Sport Coach, 20YE).*

*you have to make a connection with them …..these people are unemployed, they’ve got housing problems, they’re not working, and also they’ve got addicted to something… you have to sit down with them….discuss with them. It’s someone they can listen to… (M, Community Leader and Sport Coach, 5YE).*

Specifically, public involvement was identified as a way to enable sport coaches to recognise diversity and inequality and its impact on physical activity:

*(Hounslow) is more diverse. Not everyone in a group goes to the local community centre for activity …not all Asian groups are the same. We need ways of understanding people a bit better …what are some of the conflicts they’re having…so we can offer solutions to (health) problems and not give them another thing they have to do (F, Community Sport Commissioner, 10YE)*

Sport coaches supported the use of community focus groups, ‘meet the coach’ and taster sessions as effective forms of public involvement. These activities were important in facilitating active participation of potential participants and in helping community sport coachesto make the right decisions about the implementation of local community sport. Public involvement activities were viewed as a form of collective ownership of the community sport service:

*in the past, it’s just been putting on activities and hoping that people turn up if it’s marketed. That’s not attracting the right (inactive) people. For our work …..to be embedded within the communities that you want to work within requires local people to get on board….we need to get out and meet them to reach the people that’s the hard to reach or most at need … we wanted to have more of a relationship with our participants…so we can decide and act together (F, Physical Activity Project Coordinator, 3YE).*

For the sport coach workforce, working with participants was a form of community empowerment. It enabled potential participants to influence the development of community sport and physical activity programmes:

*I don’t think there’s any point you just putting something on. Present it, get feedback, discuss it and make decisions together. if people are a bit more informed and actually really understand what the drive is behind it, then everything makes more sense you know… it empowers you a little bit to think…and understand…to take ownership of a project (F, School-Community PE Specialist, 25YE).*

### Building collaborations between community sport and public health

Collaborative working between researchers, local and national sport policy makers, community sport coaches, managers and commissioners, public health professionals and participants defined the design, delivery and evaluation of the HASE project overall. The London Borough of Hounslow had an established network of community sport and physical activity partners operating through a CSPAN (Community Sport and Physical Activity Network). This forum was important to the inception and implementation of the project:

*I think the [HASE] approach suits Hounslow really well …six years ago, we were still working very much in siloes and everybody was doing their own thing. Since then we’ve had everybody working together on the community sports, this connectivity network …all our projects are about partnership work across the borough, across the range of services, and across a range of boroughs, that’s linking in and sharing expertise and resources (F, Community Sport Development Lead, 20YE).*

Two aspects of collaborative working were identified by the interviewees: knowledge exchange, and partnership approaches.

### Knowledge exchange

The opportunity for knowledge exchange between different sport coaches and public health professionals was central to successful partnerships and for sustained delivery of community sport for health outcomes:

*The knowledge exchange was powerful for me because I could see there’s a lot of opportunity …for communities to bind themselves together by way of sport, and to give those people a healthy option in order to live a better life (M, Community Sport Coach, 10YE).*

*that knowledge exchange workshop…it was the first time I came into contact with some of those people who did those various jobs. There was the health trainers…I didn’t even know they existed to be honest! So it was interesting to find out how they work with their clients and maybe if they’re looking to refer them to like organisations such as ourselves where they can do regular exercise then there’s maybe a partnership (M, Community Sport Coach, 5YE).*

The challenges of promoting public health through community sport, and developing more systematic, collaborative and larger scale working relationships between community sport and local health agencies, were recognised:

*Physical activity is a core area in public health. Sport - I think it’s very relevant …for me it’s a new role and the problem is that sports clubs can sometimes be a bit cliquey… you’ve got those added complexities…confidence…what to wear…not knowing anyone. But, having said that, you know, then sports clubs can have quite a nice social side, which gives another added dimension to people and makes them feel part of a community and to have something additional that they can engage with in a really positive way, but it’s just how that happens and how you get to that point (F, Community Sport Commissioner, 10YE).*

However, the significance of HASE planning and training activities in enabling partnership working between public health professionals and community sport coaches for health outcomes dominated the views:

*I was hesitant but I did learn a lot …I am going to work together with the Hounslow Homes project now (F, Community sport coach, 20YE).*

*I’ve now got a relationship with Integrated Neurological Services and we’re working on developing and delivering a programme (F, Physical Activity Manager and Sport Coach Commissioner, 8YE).*

*My coaches are understanding more about the health agenda and people within health are understanding more about the positivity of doing sport and physical activity as well…local connections worked really well (M, Senior Community Sport Manager and Sport Coach, 15YE).*

### Partnerships, pathways for recruitment and promoting sport for health

Partnership working in public health commonly involves strategies for bringing people together and enabling engagement, making pathways for recruitment and issues of promotion and communication key. A core ambition for commissioners, managers and sport coaches in this study was for the development of a referral system for community sport activities, based on existing referral approaches in public health that could develop a partnership between public health and community sport in working with inactive communities:

*I think that going forward we’re trying to engage a large amount of inactive people, what would work well is referrals into a programme. Self-referrals or GPs or health trainers are a key to referring to community sport. And then also there’s that knowledge exchange from sports clubs, coaching professionals and volunteers, to understand how health professionals do their work ….and help them signpost to us (M, Director, Community Health Organisation, 8YE).*

*I feel that there needs to be an agreed way forward in the whole Borough… for a referral process…recognising and going out to physical activity deliverers so if in public health you’re sitting there doing your individual target sheet with your client, they want to get fit, they have mobility issues, they’re over 60 or whatever, refer them to me, contact me (F, Physical Activity Project Coordinator, 3YE).*

Sport coaches and those involved in commissioning community sport identified established public health strategies, theoretical approaches and pathways to recruitment as relevant to the work of sport coaches:

*my starting point..for community sport …would be around NICE guidance that clearly states there’s an evidenced way of doing things…quite complex and requires a certain skill base ….but actually when people have done that work it’s a lot easier and we need sport delivery teams to know about giving advice and motivating people take part (F, Community Sport Commissioner, and Sports Coach 10YE).*

*have sport coaches understand behaviour change …advising and motivating …is quite important (M, Commissioner, 15YE).*

A more formalised role for sport coaches in engaging and supporting inactive people to become active through community sport was identified:

*you could see a role for a physical activity activator or sport champion …giving support to inactive people to get active … understanding everything, .helping decisions, signposting to relevant services (M, Director, Community Health Organisation, 8YE).*

The common theme in discussions about recruiting and signposting to community sport was the idea of moving beyond traditional health promotion messages associated with exercise prescription to a focus on knowledge and understanding about the role of sport for health and enabling people to take part:

*(we have to) avoid very old health messages about exercise as something else they have to do. I think better ways of messaging are with some of the behaviour change ways …but through understanding people so it’s more for them (F, Community Sport Commissioner, 10YE)*

## Discussion

### Principal findings

This study recognised that engaging inactive people in sport lies outside sports coaches previous experience and aimed to equip them for this new role. All interviewees agreed that formal training via the RSPH Level 2 Award was a key ingredient for increasing sports coaches’ knowledge and understanding about public health. Bespoke workshops on targeting, recruiting and retaining inactive people to community sport allowed coaches to develop their skills and knowledge and maximise the potential for raising physical activity levels in their work. Public involvement was also unanimously viewed as essential to better understanding of the barriers and facilitators to sport for diverse community groups. Moreover, it was identified as a way to understand better and attempt to resolve the complex personal and social experiences that mitigate against taking part. Engaging potential participants in the design of community sports projects was found to be important in appropriately tailoring community sport programmes. Public involvement allowed a focus on collective ownership of the content and delivery of community sport and was considered central to successful participant engagement. On-going opportunities for knowledge exchange between sport coaches and public health professionals was recognised as a pathway to sharing best practice in identifying, supporting and empowering inactive people to become more active. A more formal role for sport coaches in delivering community sport to increase levels of physical activity was articulated as important by our interviewees.

Overall, the need for partnerships between local public health and community sport sectors was advocated for successful service delivery of community sport for inactive people. Yet, the significant challenges of promoting public health through community sport were identified. Overcoming the negative perceptions of sport and addressing the problems of ensuring more systematic, collaborative and larger scale working relationships between community sport and public health organisations were identified.

### Contribution to knowledge

The findings support the work that has identified sports coaches as potential community assets, helping local communities to address public health concerns around raising levels of physical activity [12, 21]. In addition, the study adds to and develops further the conclusions of other studies which contend that sports coaches have a wider role than the teaching of sport skills to play in individual and community development of life skills [33], positive social behaviours [34], and supporting mental health [35]. While others have identified that sports coaches are not routinely trained in public health or the needs of inactive populations [21], this study shows that with the right training and partnership arrangements sports coaches have the potential to identify inactive people and engage and support them in tailored community sport programmes. They can, therefore, offer both a complementary and additional service in public health behaviour change broadly, one which is typically linked with the work of GPs or practice nurses [16, 17, 22]. The processes by which sports coaches in this study engaged and supported inactive people to take part in community sport reflects the significance of strategies reported in the wider literature which seek to develop understandings of complex and diverse personal and social experiences which make it difficult for people to participate including youth [36], age and ageing [37], disability [38], gender [39], sexual orientation [40], socio-economic status [41] and the environment [42] and crucially the intersections of such socio-cultural, environmental and individual issues. Pedagogical implications for coaches are revealed in this study. Supporting inactive people to become involved in community sport for health requires learning in practice and our findings indicate the salience of developing innovative health-related pedagogical skills and knowledge for the coaching workforce [43]. This also requires recognition of the complex reality of designing and delivering sport for inactive people and a more innovative approach to coaching; one that is not solely based on competencies but recognises the need to build, enhance and apply different skills and knowledge in reaching and engaging diverse groups of inactive people with a range of health and wellbeing needs in community sport [44]. This study illustrates the importance for researchers and practitioners, of developing theoretical and applied work that moves beyond established behaviour change approaches to physical activity to consider complex everyday relationships, and develop knowledge and understanding about the challenges of and best practice in empowering people to take part in community sport for health and wellbeing. It also illustrates the scope for drawing on work from the social sciences that provides deeper understandings of social diversity in sport and physical activity which can be applied in supporting those who find it most difficult to take part [45–48]. These findings support those from studies that have argued for public involvement in community health projects to improve service quality, programme relevance, participant engagement and satisfaction, and health outcomes [49]. The present study also reinforces the potential in co-design approaches for ensuring that the needs of all stakeholders are addressed and that there is shared ownership and responsibility for project outcomes [50, 51]. The findings support calls for workforce development, knowledge exchange and partnership approaches in the community sport sector to reflect public health concerns connected to raising physical activity levels [22, 52]. It is emphasised that there remain challenges in overcoming negative perceptions of sport and in scaling up public health and community sport partnerships for population level change in physical activity.

### Strengths and weaknesses

#### Strengths

The study was part of a rigorous mixed methods study design for which there is a published protocol. The use of one interviewer provided some consistency in questioning and the sample included a diverse range of stakeholders centrally involved in community sport coaching or the management and commissioning of coaching. Interviews were conducted by the project lead who was involved in other aspects of the research providing some consistency and a systematic approach to data collection and analysis throughout the project.

#### Weaknesses

The sample was self-selecting which can create some bias in the data. One coach did not attend the knowledge exchange workshop due to other work-related commitments and may have had an experience and views on that aspect of the training which could have affected the findings.

### Implications for practice and research

Sport coaches have a role in designing and delivering complex community sport interventions for increasing physical activity and improving health. However, there is a need to understand how the knowledge and skill set of this workforce can be advanced for them to realise their potential as community resources in public health. This study has identified that it is possible to build capacity for delivering sport for health programmes by training sports coaches in public health, building locally specific knowledge about inactive communities through public involvement strategies, facilitating cross-sector knowledge exchange and encouraging partnership working between sport and public health sector experts. There is an increasing focus on community sport delivery for public health outcomes. Such delivery is complex and there is a need for research to focus on developing the evidence base on the processes involved in sport coach delivery and the impact of sport coaches on the successes, impacts and outcomes of interventions to support intervention design. It is equally important that findings of such research are disseminated in useful and useable ways, and in varied forms to different stakeholders and user groups so they can capitalise on and use the evidence in their policy and practice work.

## Conclusions

Complex community sport interventions have the potential to engage inactive people to increase physical activity for health. Such interventions are likely to be delivered by sport coaches whose knowledge and expertise in public health and recruiting inactive people to sport is partial. This study has shown that with the right training, sport coaches can develop and apply their knowledge and understanding of public health and their skills in targeting, recruiting and retaining inactive people to community sport. The findings emphasise the importance of public involvement in supporting engagement of inactive people in community sport. In addition, the study has shown that through knowledge exchange between the community sport and public health sectors, there is potential for reciprocal partnership arrangements to develop that could further equip sport coaches with the knowledge and skills to design and implement community sport and potentially develop population level interventions for increasing physical activity, reducing inactivity and improving public health.

## Additional file

Additional file 1: Agenda of interview questions (*and prompts)*. (DOCX 14 kb)

## Abbreviations

CSPAN: Community Sport and Physical Activity Network
HASE: The Health and Sport Engagement Projects
LBH: London Borough of Hounslow
RSPH: Royal Society for Public Health
