# Supplementary material for: A qualitative investigation of the role of sport coaches in designing and delivering a complex community sport intervention for increasing physical activity and improving health
Source: BMC Public Health. 2018 Oct 22;18:1196. doi: 10.1186/s12889-018-6089-y (PMC6198428; doi:10.1186/s12889-018-6089-y)
Supplement: Supplementary file 1 — Agenda of interview questions (and prompts). (DOCX 14 kb) [file 12889_2018_6089_MOESM1_ESM.docx]

**A qualitative investigation of the role of sport coaches in designing and delivering a complex community sport intervention for increasing physical activity and improving health.**

**Agenda of interview questions (*and prompts)***

**1.Could you describe you job role in the community sport sector?**

*What is your job title?*

*Describe a typical day and week in your job*

*What kind of participants do you work with and in what contexts?*

*How long have you been in this job and how did you get into it?*

**2. What kind of experiences have you had in delivering sport for increasing physical activity and improving health?**

*Do you include health in the way you work with participants? How? What kind of programmes might work this way? How and why is community sport important to public health?*

*Do you work with people who are inactive? If so, could you explain the work and the participants? If not, could you see it as relevant to you work? In what ways? What are the challenges of working with inactive people in community sport? What are the benefits?*

**3.Perceptions and expectations of the HASE project**

*What were your reactions to the idea of a community sport (HASE) project for improving health? Was it relevant? Timely? Achievable? Measurable?*

*What did you expect from the HASE project?*

*How important are local community approaches to delivering sport? In what ways are they best delivered? What are the challenges?*

*What do you know of national strategies for community sport for public health? Can local projects be designed and delivered for national aims?*

**4.Views about the causes, consequences and experiences of inactivity**

*What is your view about levels of inactivity – nationally – locally? Who are the least active in this Borough?*

*Is your work relevant to inactive people? If so, in what ways?*

*How important is community sport to reducing inactivity?*

*How can we best work with people who are inactive in community sport?*

**5.What was your view about the training schedule for sports coaches?**

*What aspects of the RSPH training were most relevant? New to you? Challenging or less relevant? How might you apply what you learned in your community sport work?*

*What aspects of the design and promotion workshops were most relevant? New to you? Challenging or less relevant? How might you apply what you learned in your community sport work?*

*What aspects of the knowledge exchange activities were most relevant? New to you? Challenging or less relevant? How might you apply what you learned in your community sport work?*

**6.What about the way the training was delivered?**

*What did you find most useful about the structure and delivery of the training?*

*What did you find most challenging about the structure and delivery of the training?*

*What would you do differently in terms of the training and why?*
